# Commentary: The Circulatory Effects of Increased Hydrostatic Pressure Due to Immersion and Submersion

**DOI:** 10.3389/fphys.2022.830759

**Published:** 2022-01-27

**Authors:** Jacques Regnard, Malika Bouhaddi, Olivier Castagna, Laurent Mourot

**Affiliations:** ^1^EA3920 Prognostic Factors and Regulatory Factors of Cardiac and Vascular Pathologies, University of Bourgogne Franche-Comté, Besançon, France; ^2^Department of Physiology and Functional Testing, University Hospitals of Besançon, EA3920 Prognostic Factors and Regulatory Factors of Cardiac and Vascular Pathologies, University of Bourgogne Franche-Comté, Besançon, France; ^3^Underwater Research Team (ERRSO), Military Biomedical Research Institute (IRBA), Toulon, France; ^4^UPR 66312 Human Motricity and Sport Health Expertise Laboratory (LAHMES), Azur Coast Nice University, Nice, France; ^5^EA3920 Prognostic Factors and Regulatory Factors of Cardiac and Vascular Pathologies, Exercise Performance Health Innovation (EPHI) Platform, University of Bourgogne Franche-Comté, Besançon, France; ^6^Division for Physical Education, National Research Tomsk Polytechnic University, Tomsk Oblast, Russia

**Keywords:** immersion, gravity, hydrostatic pressure, Pascal's Law, blood reallocation, fluid movements

We noted with interest the article by Weenink and Wingelaar published last July in Frontiers in Physiology (Weenink and Wingelaar, 2021). However, reading the abstract and the article was largely disappointing. We cannot agree with many statements made by the authors. In our opinion, their analysis of the physiological responses to immersion lacks precision in physical and physiological evidence presented, and cannot support the actual mechanisms underlying their findings.

One cannot assert with certainty that buoyancy reduces movement of fluid from the vascular to extracellular compartment. Also, the authors suggest that the hydrostatic force does not exert a compressive force on the body and this is not correct. In a fluid (liquid or gas), hydrostatic pressure acts in every direction on the sheath of the immersed object (or body). Buoyancy results from the upward force experienced by the whole object according to both the volume of fluid displaced and the difference between densities of the object/body and the fluid. For example, a hot air balloon gets buoyancy from the atmospheric (hydrostatic) pressure due to the lower density of the inner volume than the same atmospheric volume outside.

However, the “Archimedes principle” operating on the entire immersed human body does not exert any buoyancy effect inside the vascular network where gravity still acts on blood exactly as when outside water. In an upright position an immersed subjects' pulmonary tissue is still less distended in the base of the lungs which supports the whole lung weight than in the apex. X-Ray evidence of immersion pulmonary œdema appears in different lung areas that relates to the subject's position during the œdema development (Hårdstedt et al., 2020; Castagna et al., 2021). Similarly, degrees of lung ventilation and perfusion are known to change with subject's position in lying patients. Further, during immersion “Archimedes buoyancy” roughly equilibrates the weight of human body, but does not suppress gravitational effects. Thus, while the “weightlessness” sensed during immersion arises from this buoyancy, the immersed cardiovascular physiology remains different from cardiovascular physiology during real microgravity in orbit. Even though thoracic blood volume is increased under both conditions, in spacecrafts no external body compression operates nor does hydrostatic pressure (Regnard et al., 2001; Prisk, 2011). Relying simply on the word “weightlessness” is confusing in the context of a purposely comprehensive article.

Hydrostatic pressure is very much a compressing force able to squeeze compliant vessels and reduce total vascular capacity, which in an emerged state amounts to nearly five times the blood volume. Blood flow and volume are allocated to various parts of the vascular network according to circumstances: resting supine or upright, leg or arm exercising… Gradually increasing immersion exposure from the feet to the neck progressively reduces total vascular capacity (Koubenec et al., 1978). The compression by hydrostatic pressure and the accompanying displacement of blood volume immediately shifts this volume upon squeezing the leg and trunk vessels inducing enlarged heart chambers and an increased central venous pressure (Risch et al., 1978; Johansen et al., 1992, 1995, 1997). Likewise pneumatic anti-shock garments and g-suits compress and increase cardiac preload (Regnard et al., 1990; Gilbert et al., 1991). Similar reduction of vascular capacitance and displacement of blood volume are not produced when people are placed in dry hyperbaric chambers. Three bars in compressed air does not compress leg and thigh vessels nor increase heart dimension by even one tenth of a bar in water. Upright water immersion up to heart level creates a hydrostatic pressure around the ankle of e.g., 125 cm H_2_O or 90 mmHg, which roughly equals hydrostatic pressure in the body blood column (at the ankle level). In air the ankle skin is subjected to an external atmospheric pressure of 0.11 mmHg higher that at the heart level (800 times lower than the internal hydrostatic pressure), and in compressed air at a 3-bar pressure, the external atmospheric pressure at the ankle is only 0.34 mmHg higher than at heart level. Hydrostatic pressure applied to the skin is transmitted by tissues of grossly hydric density (hence little compressible as skin, muscles, liver, kidney…) at the adventitial side of vessels to exert a squeezing effect. Because room is available in other parts of the vascular network (pelvic, mesenteric, lung…), blood is shifted in more or less incompletely filled vessels. This shift of blood volume operates no matter the position, i.e., independently of gravity direction relative to the body. Quite similar pulmonary and heart engorgement occurs in upright and supine immersed subjects, as reflected in the autonomic nervous system adjustments in heart rate and vasomotor tone (Bahjaoui-Bouhaddi et al., 2000; Mourot et al., 2007, 2008). A cold-triggered vasoconstriction can be added without or with face cooling (Mourot et al., 2008).

The external hydrostatic pressure changes the transmural pressure in vessel walls, which in capillaries and venules affect microvascular filtration between the vascular and interstitial spaces. In governing these exchanges gravity controls blood pressure (hydrostatic pressure) differently from interstitial pressure (e.g., with postural changes), leading to microvascular filtration or conversely plasma reabsorption (Hagan et al., 1978). The time constant of transmural fluid exchange is much higher than that of blood displacement across vascular beds. Thus, upon immersion the rapid (about 1 s time constant) increase in thoracic blood volume results from arrival of blood from the compressed legs and thigh muscles and then from the abdominal vessels (Risch et al., 1978; Regnard et al., 1990; Johansen et al., 1997). The much slower increase in plasma volume (hemodilution) is due to reabsorption of interstitial and intracellular fluids, largely in the lower limbs (Greenleaf et al., 1980; Johansen et al., 1992, 1997; Pendergast et al., 2015). The “Perspective” article by Weeninck and Wingelaar missed the pivotal distinction between rapid blood translocation between vascular beds (Risch et al., 1978; Gilbert et al., 1991), and the much slower (and long lasting) interstitial fluid reabsorption from extravascular space that leads to the increased urine output (Johansen et al., 1992, 1997; Castagna et al., 2013).

After firstly dismissing any hydrostatic compression effect and suggesting buoyancy is at play, the authors then rely on the compressive effect of “tight fitting suits” to make their arguments. It is of note that the pressure exerted by elastic neoprene suit has been accurately measured (Castagna et al., 2013) and yes hydrostatic pressure acts as elastic stocking (Tipton et al., 2017) and can reduce microvascular filtration or induce interstitial fluid reabsorption (Johansen et al., 1997).

The analysis of immersed lung mechanics improperly refers to Pascal's Law, which operates on incompressible fluids, not on lung gas spaces. The miscellaneous discussion (section “Additional factors to consider”) is inaccurate regarding the data presently available (Wilmshurst et al., 1989; Castagna et al., 2018; Wilmshurst, 2019). It is not true that after immersed cooling a subject is in a vasoplegic state. In fact the reduction in large cold-induced arterial and venous vasoconstrictive tone occurs over several hours (Robinet et al., 2006; Boussuges et al., 2009; Florian et al., 2013; Riera et al., 2014). Yes removing the squeezing effect of hydrostatic pressure precipitate rescue collapse (Lloyd, 1992).

As presented in the paper several physical or physiological concepts are misleadingly described and some related statements are therefore uninformative and difficult to accept. Quoted as they were, these perspectives had to be cautiously considered. Much recent factual evidence is not considered. Main physiological effects of immersion are sometimes too simply explained adding confusion and bordering on misleading. All this can putatively detract from a clear understanding for the readers.

## Author Contributions

JR wrote the first draft. MB, OC, and LM added contributions and revised the manuscript. The final version was approved by all authors.

## Conflict of Interest

The authors declare that the research was conducted in the absence of any commercial or financial relationships that could be construed as a potential conflict of interest.

## Publisher's Note

All claims expressed in this article are solely those of the authors and do not necessarily represent those of their affiliated organizations, or those of the publisher, the editors and the reviewers. Any product that may be evaluated in this article, or claim that may be made by its manufacturer, is not guaranteed or endorsed by the publisher.
